# Comment on “Does the 5-2-1 criteria identify patients with advanced Parkinson’s disease? Real-world screening accuracy and burden of 5-2-1-positive patients in 7 countries”

**DOI:** 10.1186/s12883-024-03692-2

**Published:** 2024-06-05

**Authors:** Harmen R. Moes, Erik Buskens, Teus van Laar

**Affiliations:** 1 Department of Neurology, University of Groningen, University Medical Center Groningen, Groningen, 9713 GZ The Netherlands; 2 Department of Epidemiology, University of Groningen, University Medical Center Groningen, Groningen, The Netherlands

**Keywords:** Advanced Parkinson’s disease, Device-aided therapy, 5-2-1 criteria, Validation, Positive predictive value

## Abstract

The 5-2-1 criteria are intended to help general neurologists identify patients with advanced Parkinson’s disease who may benefit from treatment optimisation, such as with a device-aided therapy. Although the 5-2-1 criteria claim to address an unmet need, we urge readers to cautiously interpret the results of this validation study.

**Supplementary Information:**

The online version contains supplementary material available at10.1186/s12883-024-03692-2.

*Dear editor*,

We have read with great interest the article entitled, “Does the 5-2-1 criteria identify patients with advanced Parkinson’s disease? Real-world screening accuracy and burden of 5-2-1-positive patients in 7 countries” by Malaty et al. [1]. The authors correctly point out the lack of an objective and uniform method or tool for timely identification of patients with advanced Parkinson’s disease (PD) who are inadequately controlled on oral medication and who may benefit from treatment optimisation, such as the initiation of a device-aided therapy (DAT) [2]. The apparently user-friendly 5-2-1 criteria reviewed in the article have been proposed to meet this clinical need [2, 3]. Although these criteria are based on expert opinion (a Delphi study) and have not been developed according to accepted scientific guidelines for multivariable models, they might still be fit for purpose [4, 5].

The authors present the results of a validation study of the 5-2-1 criteria in a cohort of 4714 patients from 7 different countries [1]. However, we are concerned about some aspects of the methodology and the non-reporting of less favourable test characteristics, as we will discuss below. We will also cover the response to our comments by Antonini et al. [6].

First, the composition of the study population might have resulted in overestimation of the 5-2-1 criteria’s performance measures. For accurate validation, a model should be evaluated in a setting that reflects its intended use [5]. The 5-2-1 criteria are intended for use by general neurologists who may typically lack expertise in identifying patients with advanced PD eligible for DAT referral. However, the population in this validation study, comprised of PD patients naive to DAT, had a higher prevalence of advanced PD (14.9%) compared to that observed in general neurological practice (6.7% in our own study) [7]. It is important to note that a high prevalence within a validation population will generally lead to overly optimistic estimates of both the positive predictive value (PPV) and sensitivity of the 5-2-1 criteria, particularly when compared with estimates derived from populations with lower prevalence, such as those seen in a general neurological practice [8, 9].

Second, in this validation study, the reference test (gold standard) was based on a single neurologist’s assessment of each patient’s disease severity [1]. This approach reflects the particular setting of the current study, i.e., the evaluating neurologists had substantial expertise in identifying patients with advanced PD. However, if we assume that the study population is representative of a general neurological practice, it is unlikely that all evaluating neurologists have such extensive experience in assessing advanced PD. This discrepancy could have led to outcome misclassification in the validation study (i.e. patients being misclassified by the ‘gold standard’ evaluating neurologist as having or not having advanced PD) [10]. A possible method to reduce such bias is to use a consensus of multiple experts as the gold standard [7, 11].

Third, the accuracy of the 5-2-1 criteria was misreported. The researchers define the correct classification rate (CCR) as the sum of true positives and true negatives divided by the total number of patients [1]. However, the percentages in the table on page 5 of the article by Malaty et al. do not correspond to the figures in the cross-tabulation shown [1]. In our Table 1, we have presented the tabular data from the article with our own calculations, which show a CCR of 75.7%, whereas the article reports 88.1%. Moreover, it is important to note that the CCR is a misleading evaluation metric in so-called imbalanced datasets [12]. For example, if the sensitivity of a test is as low as 0% in a setting with a prevalence of 14.9%, the CCR would still be 85.1% (table A1 in Appendix).

**Table 1:** Data from Table 2 of the article by Malaty et al. (2022) [1] with our own calculations of the accuracy measures. It should be noted that we inverted the columns and rows of the cross table, so that “yes” and “positive” appear on the left and top of the cross table, respectively. In the cross table, the letters A, B, C and D are shown to indicate the calculation steps used to calculate the diagnostic measures

		advanced PD			Malaty et al.	Our calculations of accuracy measures			
**5-2-1 screening criteria**		**yes**	**no**	^∑^	**CCR** ^** (A+D)/(A+B+C+D)**^		**SENS** ^**A/(A+C)**^	**SPEC** ^**D/(C+D)**^	**PPV** ^**A/(A+B)**^
	**pos**	552^**A**^	994^**B**^	1546	88.1%	75.7%	78.6%	75.2%	35.7%
	**neg**	150^**C**^	3018^**D**^	3168					
	^**∑**^	702	4012	4714					
**≥ 2 h off-time/day**		**yes**	**no**	^**∑**^	88.4%	80.5%	70.4%	82.2%	40.9%
	**pos**	494^**A**^	713^**B**^	1207					
	**neg**	208^**C**^	3299^**D**^	3507					
	^**∑**^	702	4012	4714					
**≥ 1 h troublesome dyskinesia/day**		**yes**	**no**	^**∑**^	87.1%	86.0%	15.8%	98.3%	62.0%
	**pos**	111^**A**^	68^**B**^	179					
	**neg**	591^**C**^	3944^**D**^	4535					
	^**∑**^	702	4012	4714					
**≥ 5 doses of oral levodopa/day**		**yes**	**no**	^**∑**^	87.1%	80.4%	41.2%	87.3%	36.1%
	**pos**	289^**A**^	511^**B**^	800					
	**neg**	413^**C**^	3501^**D**^	3914					
	^**∑**^	702	4012	4714					

Abbreviations: CCR = correct classification rate, SENS = sensitivity, SPEC = specificity, PPV = positive predictive value, pos = positive, neg = negative, ∑ = row/column total.

Finally, some less favourable test characteristics of the 5-2-1 criteria were not reported (Table 1) [1]. The authors chose to report the area under the curve (AUC) values, rather than also including the sensitivity (78.6%) and specificity (75.2%). As a summary metric, the AUC does not provide an immediate insight into the clinical implications of using the 5-2-1 criteria [13]. Similarly, the authors did not discuss the implications of the low PPV of 35.7%, which suggests that a significant proportion of 5-2-1-positive patients may not yet have advanced PD according to the reference test [1]. This low PPV implies that application of the 5-2-1 criteria could lead to many patients being classified as having advanced PD, potentially leading to premature referral for DAT and consequently an increased burden on the referral network.

Our outlined concerns about the validation study of the 5-2-1 criteria have been addressed by Antonini et al. [6] (published elsewhere in this journal). In their response, Antonini et al. also provide additional accuracy measures for both the unadjusted 5-2-1 screening criteria and the adjusted regression model of these criteria. The authors seem to claim that the adjusted model reflects the true performance of the 5-2-1 criteria. However, below we argue that only the unadjusted analysis should be considered.

For the adjusted regression model of the 5-2-1 criteria, the apparent higher PPV comes at the cost of a much lower sensitivity of 41.9%. The authors erroneously state that a low false negative rate (FNR) was maintained. However, the FNR here refers to patients with advanced PD who are not identified by the 5-2-1 criteria and is calculated as 100 - sensitivity. In the adjusted model, the FNR is 58.1%, which we consider to be very high. Contrary to the author’s claim, the negative predictive value (NPV) is not a good indicator of the FNR, as the NPV depends on the prevalence [14]. While Antonini et al. argue that the 5-2-1 criteria would reduce under-referral, they neglect the implications of the low sensitivity of the adjusted model.

Because the adjusted model reported by Antonini et al. has different accuracy measures than the unadjusted analysis, we reconstructed the crosstabs (Table 2). The question then becomes, which crosstab reflects the true screening performance of the 5-2-1 criteria? The adjusted model with low sensitivity but high specificity and PPV, or the unadjusted 5-2-1 criteria with reasonable sensitivity but lower specificity and low PPV?

**Table 2:** Cross table based on the accuracy measures as provided in the response by Antonini et al. [6]. The calculation steps for creating the cross table of the adjusted model are described at the bottom of the table

		advanced PD			CCR	SENS	SPEC	PPV	NPV
**adjusted regression model of 5-2-1 criteria**		**yes**	**no**	**∑**	88.1%	41.9%	96.2%	65.9%	90.4%
	**pos**	294†	152§	446					
	**neg**	408¥	3860*	4268					
	**∑**	702	4012	4714					
**unadjusted** **5-2-1 screening criteria**		**yes**	**no**	**∑**	75.7%	78.6%	75.2%	35.7%	95.3%
	**pos**	552	994	1546					
	**neg**	150	3018	3168					
	**∑**	702	4012	4714					

Abbreviations: CCR = correct classification rate, SENS = sensitivity, SPEC = specificity, PPV = positive predictive value, NPV = negative predictive value, pos = positive, neg = negative, ∑ = row/column total.

† the number of true positives was calculated as follows: sensitivity × total number of patients with advanced PD (41.9% x 702 = 294).

¥ the number of false negatives was calculated as follows: total number of patients with advanced PD – true positives (702–294 = 408).

* the number of true negatives was calculated as follows: specificity x total number of patients without advanced PD (96.2% x 4012 = 3860).

§ the number of false positives was calculated as follows: total number of patients without advanced PD – true negatives (4012–3860 = 152).

The adjusted model was constructed using multivariable logistic regression to adjust for potential confounders, such as country, age and gender [1]. Importantly, adjustment for confounding was unnecessary because confounding is only an issue in research on causal relationships, not in prediction research such as screening studies [10, 15]. In addition, the authors did not present the full regression model including the intercept and regression coefficients, making it impossible for the reader to apply the model to an individual patient of a particular gender, age and nationality [5]. Furthermore, it is unclear how the final regression model was derived, as not all modelling steps are documented [5, 13]. For example, the authors do not explain why a cut-off of 0.5 was chosen for the calculated probabilities of the adjusted model, whereas any other cut-off would result in a different ratio of sensitivity to specificity (see Appendix for more details).

We argue that the unadjusted analysis is the only correct method to assess the true performance of the 5-2-1 screening criteria. This method allows a direct assessment of the accuracy measures from the cross-tabulation data. Therefore, we maintain that the 5-2-1 criteria have acceptable sensitivity but relatively low specificity, resulting in a low PPV of 35.7%. This is consistent with our own analysis of the 5-2-1 criteria [7]. Possibly, the PPV could be increased by modification of the 5-2-1 screening criteria to require the presence of ≥ 2 criteria instead of ≥ 1.

In conclusion, the 5-2-1 criteria represent a welcome initiative flagging an unmet need. However, the validation study has several shortcomings, and the adjusted models of the 5-2-1 criteria do not provide a realistic estimate of the screening accuracy. To demonstrate the added value of the 5-2-1 criteria for real-world practice, the tool should be validated in representative PD populations, preferably following the established guidelines of STARD and TRIPOD [5, 16].

### Electronic supplementary material

Below is the link to the electronic supplementary material.

Supplementary Material 1

## Data Availability

No datasets were generated or analysed during the current study.

## Abbreviations

AUC: Area under the curve
DAT: Device-aided therapy
FNR: False negative rate
NPV: Negative predictive value
PD: Parkinson’s disease
PPV: Positive predictive value

## Declarations

### Authors’ information

HRM received the aforementioned grants for the research project “Development of a screening tool for timely referral for advanced therapy in Parkinson’s disease”. This ongoing project aims to develop and validate a clinical decision model using data from two large cohorts with prospectively collected data.

## References

[1] Malaty IA, Martinez-Martin P, Chaudhuri KR, Odin P, Skorvanek M, Jimenez-Shahed J (2022). Does the 5-2-1 criteria identify patients with advanced Parkinson’s disease? Real-world screening accuracy and burden of 5-2-1-positive patients in 7 countries. BMC Neurol.

[2] Aldred J, Anca-Herschkovitsch M, Antonini A, Bajenaru O, Bergmann L, Bourgeois P (2020). Application of the ‘5-2-1’ screening criteria in advanced Parkinson’s disease: interim analysis of DUOGLOBE. Neurodegener Dis Manag.

[3] Antonini A, Stoessl AJ, Kleinman LS, Skalicky AM, Marshall TS, Sail KR, et al. Developing consensus among movement disorder specialists on clinical indicators for identification and management of advanced Parkinson’s disease: a multi-country Delphi-panel approach. Curr Med Res Opin. 2018;34:2063–73. 10.1080/03007995.2018.1502165 30016901

[4] Moes HR, Buskens E, van Laar T (2022). Letter to the editor, validation and clinical value of the MANAGE-PD tool: a clinician-reported tool to identify Parkinson’s disease patients inadequately controlled on oral medications. Parkinsonism Relat Disord.

[5] Collins GS, Reitsma JB, Altman DG, Moons KGM (2015). Transparent reporting of a multivariable prediction model for individual prognosis or diagnosis (TRIPOD): the TRIPOD StatementThe TRIPOD Statement. Ann Intern Med.

[6] Antonini A, Chaudhuri KR, Domingos J, Jimenez-Shahed J, Wright J, Yan CH, Alobaidi A, Bergmann L, Onuk K, Harmer L and Malaty IA. Response to letter to the editor regarding “Does the 5-2-1 criteria identify patients with advanced Parkinson’s disease? Realworld screening accuracy and burden of 5-2-1-positive patients in 7 countries”. BMC Neurol. 2024. 10.1186/s12883-024-03691-3. 10.1186/s12883-024-03691-3 PMC11290257 39080589

[7] Moes HR, ten Kate JM, Portman AT, van Harten B, van Kesteren ME, Mondria T et al. Timely referral for device-aided therapy in Parkinson’s disease. Development of a screening tool. Parkinsonism Relat Disord. 2023;109. 10.1016/j.parkreldis.2023.105359 36958065

[8] Leeflang MMG, Rutjes AWS, Reitsma JB, Hooft L, Bossuyt PMM. Variation of a test’s sensitivity and specificity with disease prevalence. Can Med Assoc J. 2013;185:E537 LP-E544. 10.1503/cmaj.121286 PMC3735771 23798453

[9] Reilly BM, Evans AT (2006). Translating Clinical Research into Clinical Practice: impact of using prediction rules to make decisions. Ann Intern Med.

[10] Grobbee DE, Hoes AW. Clinical epidemiology: principles, methods, and applications for clinical research. Jones & Bartlett; 2014.

[11] Bertens LCM, Broekhuizen BDL, Naaktgeboren CA, Rutten FH, Hoes AW, van Mourik Y et al. Use of Expert panels to define the Reference Standard in Diagnostic Research: a systematic review of published methods and reporting. PLoS Med. 2013;10. 10.1371/journal.pmed.1001531 PMC3797139 24143138

[12] Akosa JS (2017). Predictive accuracy: a misleading performance measure for highly imbalanced data. SAS Glob Forum.

[13] Harrell FE. Regression modeling strategies: with applications to linear models, logistic regression, and survival analysis. 2nd edition. Springer; 2015.

[14] Best Practices B. Diagnostic test studies: assessment and critical appraisal.https://bestpractice.bmj.com/info/toolkit/learn-ebm/diagnostic-test-studies-assessment-and-critical-appraisal/. Accessed 7 Dec 2022.

[15] Ramspek CL, Steyerberg EW, Riley RD, Rosendaal FR, Dekkers OM, Dekker FW (2021). Prediction or causality? A scoping review of their conflation within current observational research. Eur J Epidemiol.

[16] Bossuyt PM, Reitsma JB, Bruns DE, Gatsonis CA, Glasziou PP, Irwig L et al. STARD 2015: an updated list of essential items for reporting diagnostic accuracy studies. BMJ Br Med J. 2015;351. 10.1136/bmj.h5527 PMC4623764 26511519

