# Correction: Comment on “Does the 5-2-1 criteria identify patients with advanced Parkinson’s disease? Real-world screening accuracy and burden of 5-2-1-positive patients in 7 countries”

**DOI:** 10.1186/s12883-024-03743-8

**Published:** 2024-07-10

**Authors:** Harmen R. Moes, Erik Buskens, Teus van Laar

**Affiliations:** 1 Department of Neurology, University of Groningen, University Medical Center Groningen, Groningen, 9713 GZ The Netherlands; 2 Department of Epidemiology, University of Groningen, University Medical Center Groningen, Groningen, The Netherlands

**Correction: BMC Neurol 24, 189 (2024)**

10.1186/s12883-024-03692-2

Following publication of the original article [1], the authors reported that the reference details to Antonini et al., 2024 (the other commentary in Matters Arising) are not correct. The correct details of the reference should be “Antonini A., Chaudhuri K.R., Domingos J., Jimenez-Shahed J., Wright J., Yan C.H., Alobaidi A., Bergmann L., Onuk K., Harmer L. and Malaty I. A. Response to letter to the editor regarding “Does the 5-2-1 criteria identify patients with advanced Parkinson’s disease? Real-world screening accuracy and burden of 5-2-1-positive patients in 7 countries”. BMC Neurology. 2024. 10.1186/s12883-024-03691-3”.

The original article [1] has been updated.
