# Supplementary material for: Comment on “Does the 5-2-1 criteria identify patients with advanced Parkinson’s disease? Real-world screening accuracy and burden of 5-2-1-positive patients in 7 countries”
Source: BMC Neurol. 2024 Jun 5;24:189. doi: 10.1186/s12883-024-03692-2 (PMC11151576; doi:10.1186/s12883-024-03692-2)
Supplement: Supplementary file 1 — Supplementary Material 1 [file 12883_2024_3692_MOESM1_ESM.pdf]

Appendix

Correct classification rate potentially misleading
The correct classification rate (CCR) can be a potentially misleading metric of screening performance [1]. In the validation study of the 5-2-1 criteria, the CCR would still be at least 85% even if all patients were classified as not having advanced PD (Table A1). In short, a high correct classification rate alone does not necessarily mean that the 5-2-1 criteria are fit for their purpose.

Adjusted model versus unadjusted analysis
The 5-2-1 criteria cannot simultaneously have the screening accuracy of both the adjusted and unadjusted model. However these models are used interchangeably in the validation study [2]. For example, the accuracy measures of the adjusted model are shown (AUC and CCR), while the text refers to the true positives and false negatives from the crosstab of the unadjusted analysis. This may be confusing to the unsuspecting reader and seems inconsistent, given the plausible assumption that the unadjusted and adjusted analyses cannot be true at the same time.

Cut-off for probabilities in the adjusted regression model
A regression model depends on several decisions and modelling steps [3, 4]. In the validation study of the 5-2-1-criteria, the calculated probabilities of the adjusted model were dichotomised using a cutoff of 0.5 to classify patients as having advanced PD or not (see Antonini et al). There was no clear explanation for the choice of this cutoff other than that it was based on widely accepted thresholds [2]. Theoretically, however, the ROC curve of the adjusted model of the 5-2-1 criteria, with an area under the curve (AUC) of 0.89, can have multiple cutoff points at which the correct classification rate remains 88.1%, while the values of sensitivity and specificity vary considerably across the spectrum of possible cutoff points (Figure A1).

Table A1 The correct classification rate at a sensitivity of 0%. The table shows the values for diagnostic accuracy if all patients would be classified as "non-advanced PD". Even though the sensitivity is 0%, the correct classification rate is still higher than 85%.

		advanced PD			CCR*	SENS	SPEC	PPV	NPV
		yes	no	Σ					
all cases classified as not having advanced PD	pos	0	0	0	85.1%	0.0%	100%	n.a.	85.1%
	neg	702	4012	4714					
	Σ	702	4012	4714					

Abbreviations: CCR = correct classification rate, SENS = sensitivity, SPEC = specificity, PPV = positive predictive value, NPV = negative predictive value, pos = positive, neg = negative, n.a. = not applicable (divided by 0), Σ = row/column total.
* Correct classification rate is calculated as the sum of true positives and true negatives divided by the total number of patients.

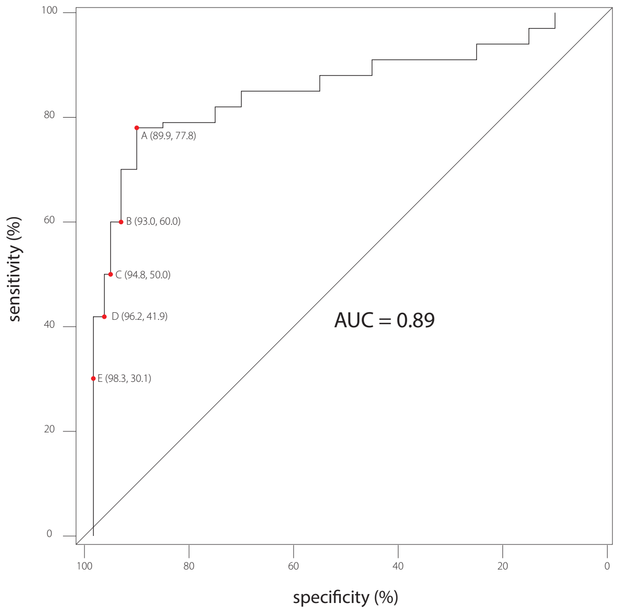

cut-off point		outcome			CCR	SENS	SPEC	PPV	NPV
A		yes	no	Σ	88.1%	77.8%	89.9%	57.4%	95.9%
	pos	546	405	951					
	neg	156	3607	4268					
	Σ	702	4012	4714					
B		yes	no	Σ	88.1%	60.0%	93.0%	60.0%	93.0%
	pos	421	281	702					
	neg	281	3731	4012					
	Σ	702	4012	4714					
C		yes	no	Σ	88.1%	50.0%	94.8%	62.7%	91.6%
	pos	351	209	560					
	neg	408	3860	3168					
	Σ	702	4012	4714					
D		yes	no	Σ	88.1%	41.9%	96.2%	65.9%	90.4%
	pos	294	152	447					
	neg	408	3860	4267					
	Σ	702	4012	4714					
E		yes	no	Σ	88.1%	30.1%	98.3%	75.6%	88.9%
	pos	211	68	280					
	neg	491	3944	4434					
	Σ	702	4012	4714					

Abbreviations: CCR = correct classification rate, SENS = sensitivity, SPEC = specificity, PPV = positive predictive value, NPV = negative predictive value, pos = positive, neg = negative, Σ = row/column total.

Figure A1 A hypothetical ROC curve with an AUC of 0.89. The figure shows five red dots (A - E) indicating different cutoff points on the ROC curve. The coordinates in the table (specificity, sensitivity) are shown. The cutoff points A - E all have a correct classification rate of 88.1% , but different values for sensitivity and specificity (shown in the table to the right of the figure). The sensitivity and specificity of cutoff point D correspond to the values reported in the reply by Antonini et al. Note: This figure is not based on the data from the study by Antonini et al., but a merely hypothetical figure to illustrate our argument.

References in this appendix

1. Akosa JS. Predictive accuracy: A misleading performance measure for highly imbalanced data. SAS Glob Forum. 2017;942:1–12.
2. Malaty IA, Martinez-Martin P, Chaudhuri KR, Odin P, Skorvanek M, Jimenez-Shahed J, et al. Does the 5–2-1 criteria identify patients with advanced Parkinson's disease? Real-world screening accuracy and burden of 5–2-1-positive patients in 7 countries. BMC Neurol. 2022;22:1–13.
3. Harrell FE. Regression modeling strategies: with applications to linear models, logistic regression, and survival analysis. 2nd edition. Springer; 2015.
4. Collins GS, Reitsma JB, Altman DG, Moons KGM. Transparent Reporting of a multivariable prediction model for Individual Prognosis Or Diagnosis (TRIPOD): The TRIPOD StatementThe TRIPOD Statement. Ann Intern Med. 2015;162:55–63.
